# Seven unique frequency profiles for scoring vigilance states in preclinical electrophysiological data

**DOI:** 10.3389/fnins.2025.1488709

**Published:** 2025-04-30

**Authors:** Freja Gam Østergaard, Martien J. H. Kas

**Affiliations:** Groningen Institute for Evolutionary Life Sciences, University of Groningen, Groningen, Netherlands

**Keywords:** electrophysiology, vigilance state, scoring, neurexin-1, slow-wave sleep, mice

## Abstract

Manual scoring of longitudinal electroencephalographical (EEG) data is a slow and time-consuming process. Current advances in the application of machine-learning and artificial intelligence to EEG data are moving fast; however, there is still a need for expert raters to validate scoring of EEG data. We hypothesized that power-frequency profiles are determining the state and ‘set the framework’ for communication between neurons. Based on these assumptions, a scoring method with a set frequency profile for each vigilance state, both in sleep and awake, was developed and validated. We defined seven states of the functional brain with unique profiles in terms of frequency-power spectra, coherence, phase-amplitude coupling, α exponent, functional excitation-inhibition balance (fE/I), and aperiodic exponent. The new method requires a manual check of wake–sleep transitions and is therefore considered semi-automatic. This semi-automatic approach showed similar α exponent and fE/I when compared to traces scored manually. The new method was faster than manual scoring, and the advanced outcomes of each state were stable across datasets and epoch length. When applying the new method to the *neurexin-1α* (*Nrxn1α*) gene deficient mouse, a model of synaptic dysfunction relevant to autism spectrum disorders, several genotype differences in the 24-h distribution of vigilance states were detected. Most prominent was the decrease in slow-wave sleep when comparing wild-type mice to *Nrxn1α*-deficient mice. This new methodology puts forward an optimized and validated EEG analysis pipeline for the identification of translational electrophysiological biomarkers for brain disorders that are related to sleep architecture and E/I balance.

## 1 Introduction

There has been an increasing interest in using electroencephalography(EEG)-based biomarkers for brain disorders, because of its robustness and high translational value (Kopell et al., 2014; Newson and Thiagarajan, 2019). For example, recordings of spontaneous electrophysiological activity are considered useful as a diagnostic marker for various neurological conditions (Voytek and Knight, 2015). However, scoring electrophysiological data into vigilance states is a tedious and slow task to perform. In this study, we propose a model for semi-automatic scoring based on frequency profiles. The hypothesis is that these power-frequency profiles are determining for the state and ‘set the framework’ for the communication between neurons.

Most modern solutions use some form of supervised machine learning to define the states for each subject (Allocca et al., 2019; Barger et al., 2019). This is typically done by manually scoring a number of epochs, based on either the filtered signal or a frequency profile. However, since the alpha rhythm (~10 Hz) was discovered in humans by Hans Berger in 1929 (İnce et al., 2021), multiple studies in various species have shown similar activity; therefore, manually training a new model for each dataset may compare to reinventing the wheel for each study. This may not only be unnecessary but also introduce variance if two states are hard to distinguish by assessing the signal visually. In addition, the currently available scoring approaches are typically very specific to certain physiological conditions, such as sleep or epilepsy, and still human expert raters are needed to validate the scoring (Fürbass et al., 2020). An advantage of employing a rule-based script is that it consistently provides the same patterns with the same labels in every dataset.

The awake substates have in general not been characterized as well as sleep states possibly because most human EEG recordings are done in seated subjects, to avoid movement artifacts. Here, we define three substates of wakefulness: a1; delta-dominated, a2; theta-dominated, and a3; alpha-dominated, named after the classically defined power bands: delta (0.5–4 Hz), theta (4–8 Hz), and alpha (8–12 Hz). Delta-dominated (a1) wakefulness has been linked to locomotor behavior (Schultheiss et al., 2020). Theta-dominated (a2) awake has been linked to active navigation, exploratory behaviors (Schultheiss et al., 2020), narcolepsy (Vassalli and Franken, 2017), and attention (Fiebelkorn and Kastner, 2019). The theta activity is thought to originate in the hippocampus (Nuñez and Buño, 2021). Similarly, delta oscillations are expected to originate in deeper structures, while the alpha is considered to be a cortical frequency. Alpha-dominated activity (a3) is most commonly referred to as resting state (Buzsáki, 2006)—the alpha oscillation is a characteristic of the idling sensory system (Kropotov, 2016). Non-rapid eye movement (nREM) sleep states are divided by their relative delta power. In human sleep-staging, there is an expectation of a fast ‘descend’ from wakeful to slow-wave sleep (Buzsáki, 2006; Genzel et al., 2014). This transition looks quite instant in rodents.

The method of rule-based scoring presented here was validated in four different and independent datasets not only by comparing the number of epochs to manually scored data but also by comparing the α exponent (α). This exponent is a measure of criticality of the signal (Stadnitski, 2012; Bruining et al., 2020) and it was added to evaluate the differences between the manual and semi-automatic scoring, as solely using the number of epochs in each state is not very descriptive of the consequences of inaccurate labeling. α is better for encapsulating how those consequences of automatic scoring may impact further analysis.

To illustrate the value of scoring all wake and sleep states, the Nrxn1α-deficient mouse model was employed. The *Nrxn1* gene encoding neurexin-1α has been associated with various psychiatric diagnosis such as autism spectrum disorder and schizophrenia (Ishizuka et al., 2020). These psychiatric disorders have been characterized by changes in sleep architecture (Phillips et al., 2012; Devnani and Hegde, 2015; Léger et al., 2018) and sensory processing (Yaguchi and Hidaka, 2020). Neurexin is a large, cell-adhesion molecule (Craig and Kang, 2007), facilitating the assembly of presynapses in early development (Dean et al., 2003). The gene expression peaks right around birth and then declines very early in life (Jenkins et al., 2016). Neurexin has been shown to regulate nighttime sleep in *Drosophila* by reducing the synaptic transmission of specific neurons (Tong et al., 2016). Here, we showed how knocking out the gene influences the distribution of vigilance states across 24-h recordings.

## 2 Materials and methods

### 2.1 State definitions for semiautomatic scoring (SAS)

The script presented here is written in MATLAB and is based on source code from AccuSleep (Barger et al., 2019) (available on Github) and the Chronux toolbox (http://chronux.org/). It uses a fast Fourier transform (FFT) to generate a frequency power spectrum for each 1 s epoch continuously sampled. Frequency profiles of the states are considered ‘fixed’ within a spectrum, while the noise levels are considered varying and are therefore adjusted manually. The number and definitions of states was based on literature and further testing if all data would fall into the defined states.

Here, three substates of wakefulness are defined by their dominating frequency band. The most common awake substate is delta awake (Schultheiss et al., 2020), defined as a bimodal pattern, with a peak in the delta range and one in the alpha range, and it is further divided into a1 and a3 depending on which range has the highest power, measured as a sum across the range rather than the power at a specific peak frequency. In a1 the delta band has the highest power, while in the a3 the alpha band has the highest power. In addition to the high power in the alpha band, the mice are inactive in the a3 state but have a high muscle tone in the neck muscles suggesting they are awake. Inactivity and high alpha matches the resting state definitions (Buzsáki, 2006); however, it is grouped with the awake states and not as the first stage of sleep as Buzsáki does. This is due to the muscle tone being higher during a3 than any of the sleep states. The a2 or theta awake (Van Gelder et al., 1991; Vassalli and Franken, 2017) is defined by a unimodal peak in the theta range. This state is independent of locomotor activity, although hippocampal theta has been associated with active navigation (Schultheiss et al., 2020). The muscle tone and theta peak frequency separates a2 from the REM sleep as the tone is higher during a2 than during REM, where the muscles are relaxed (show a low muscle tone), while the theta peak frequency is typically lower during a2, than during REM.

The non-REM (nREM) states are not following the American Academy of Sleep Medicine (AASM) scoring manual as the manual is focused on humans and a few of their definitions include non-translational measures, e.g., eye movements in N1 (Troester et al., 2023). The AASM scoring manual is based on scoring from raw traces rather than on the basis of spectrograms. In the present method, K complexes are not being taken into account here as they are not detectable in frequency-power spectra. Sleep spindles (transient activity at 11–16 Hz) affect the spectrum and are included. The nREM substates use the relative definitions: nREM1 is defined as an epoch with a power in the <10 Hz band of more than 20 times the power in the band 20–24 Hz and corresponds to slow-wave sleep (Léger et al., 2018), in nREM2 the power in the lower band is 10–20 times the power of the higher band, and in nREM3 the power of the lower band is >10 times the power of the higher band. These substates typically occur in the succession nREM1 ➔ nREM2 ➔ nREM3 ➔ nREM2/REM (see Supplementary Figure S1 for diagram).

The state definitions used here only use low-frequency activity. This has to do with the notion that higher frequency oscillations are thought to be a characteristic of localized processing (Kopell et al., 2014), suggesting that lower frequency oscillations could be characteristic of global changes in brain state.

The definitions here only require the electrophysiological signal from cortex. Motion sensors and electromyograms (EMGs) are helpful for finding transitions between wake and sleep, especially if the signal is noisy, but they are not necessary for the scoring. The application of the script is described below in Section 2.2.4.

### 2.2 Data and animals

Four datasets were used for validating the scoring. These datasets contained data from the FMR1 mouse model (males) with drugs (Kat et al., 2024), C57Bl/6J animals (9 male and 10 female, 16 weeks of age), *Pcdh9* gene knockout animals (Bruining et al., 2015) (35 males, 8 and 22 weeks of age) (Supplementary material), and *Nrxn1* animals (41 males, 10 and 22 weeks of age) (Supplementary material). In addition, 23 male *Nrxn1* mice were used for the 24-h recordings (wild-type (WT); n = 5, heterozygous gene knock-out (Het); n = 8, homozygous gene knock-out (Hom); n = 9).

Data are available on GIN with the following DOIs: Pcdh9; 10.12751/g-node.5l85fj, Nrxn1; 10.12751/g-node.j2h8vr, C57Bl/6J; 10.12751/g-node.h4ddn6.

All experiments were performed in accordance with National and EU legislation, directive 2010/63/EU of the European Parliament. All animals were housed under a 12-h/12-h light/dark cycle (lights off at 14:00), with access to water and standard chow *ad libitum*. Animal welfare was monitored daily. The animals were group housed until surgery. After surgery, they were housed in pairs with a separator that allowed for touch and smell but prevented damage to their implant. The cages were enriched with a red, plexiglass hideout and nesting material. Females and males were housed in separate rooms throughout the duration of the study. The individual weight of the mice was monitored every other week.

#### 2.2.1 Surgery

At 6 weeks of age, 23 male mice [from *Nrxn1* line (Südhof, 2017)] were anaesthetized using isoflurane (induced at 5%, maintained at ~1%) in a mix of NO_2_ and O_2_ in a 1:2 relation and administered at a flow of 1 l/min. Carprofen 5 mg/kg was administered subcutaneously (SC) in the flank, and lidocaine was injected SC on the head. The animal was placed in a robotic stereotactic frame (Neurostar, Germany). An incision was made over the skull. Then, the skull was scratched with a scalpel and prepared with 35% phosphoric acid. Craniotomies for the recoding electrodes were drilled over the visual cortex AP -4 ML ± 2.5, the prefrontal cortex AP 2.58 ML − 1.57, and the auditory cortex AP -2.5 ML ± 3.5. The reference electrode was placed over cerebellum AP -6 ML 0. Electrodes were placed on the right prefrontal cortex and bilaterally on the visual cortex and auditory cortex. A reference electrode was placed in the cerebellum, and two stranded electrodes were placed under the neck muscles to record electromyograms.

The wires for the electrodes were preassembled (ND associates, UK) into a plastic pedestal (Omnetics Connector Corporation, USA). The electrodes were jeweler screws 0.7 mm (Antrin, USA) traversing the skull and measuring the electrical potential on the cortex of the brain. The implant was attached to the skull using dental cement (3 M, USA). The skin was closed around the cement with sutures. Carprofen was administered SC at 5 mg/kg the day after surgery, and the animal was granted 2 weeks of recovery before any recordings were carried out.

#### 2.2.2 Recordings

All 4-h recordings were carried out in the late light phase, with the wireless TaiNi system (Jiang et al., 2017). The animals were freely moving during the recording sessions. The sampling rate was 1084.7 Hz, the system utilizes an online low-pass filter at 9700 Hz and high-pass filter at 0.35 Hz. The 24-h recordings of the Nrxn1s were carried out at a sampling frequency of 454 Hz, to limit the processing power required of the computer. Custom-made infrared motion sensors were placed over the recording cage throughout the recording. These motion sensors detected ~80% of all motion in the cage.

#### 2.2.3 Manual scoring

The data from the FMR1 model were manually scored by two people using the SleepScore 705 package in Spike2 v10 (CED, UK), with epoch lengths of 5 s, continuously sampled. The data were scored with a particular focus on the accuracy of scoring the resting state (a3) also called inactive wakefulness. Comparisons between scoring methods were carried out with this in mind.

#### 2.2.4 Semi-automatic scoring (SAS)

All datasets were preprocessed and passed through the SAS script (can be found on Github, https://github.com/FrejaGam/EEGcode). The script downsamples the data to make visualization of the scoring less computationally intense. If EMGs are used, a change point analysis is carried out on a sum plot of the signal to determine when the muscle tone is high (tense) and when the muscle tone is low (relaxed). These segments are then assigned to awake/tense and to sleep/relaxed. This part is sensitive to noise in the signal, and it is useful to visually assess whether the true transitions have been detected.

From here on, the overall states are subdivided automatically according to the definitions described in Section 2.1. The default epoch length is 1 s (the FMR1 study uses epochs of 0.5, 1, and 5 s). All epochs are continuously sampled.

Once the scoring is complete, a column vector of labels is created. These labels are given in ASCII with the current configuration: a; a1, b; a2, c; a3, l; REM, m; nREM1, n; nREM2, o; nREM3, N; noise, U; unscored. U is useful for development but should not appear in the final list. From the list of labels, the filtered (not downsampled) data can be distributed into states for further processing, e.g., coherence, phase-amplitude coupling (PAC), detrended fluctuation analysis (dfa), functional excitation-inhibition ratio (fE/I), and fitting oscillations and 1/f (FOOOF).

SAS was applied to data from the C57Bl/6J, *Pcdh9,* and *Nrxn1* mice to validate the stability of state definitions between datasets.

##### 2.2.4.1 Analysis of labels

The labels alone provided information on the proportion of time spent in each state along with the sequence of states and transitions between. The label data were stacked into barplots in MATLAB, using colors from the Wes Anderson Palettes.^1^ Correlation analysis was carried out in RStudio 2023.03.0 Build 386.

Sequence and transitions were inferred from epoch pairs of consecutive epochs. Combinations of the ASCII labels (not including U) yield 64 label pairs. Stacked bar plots are shown in Supplementary Figure S8, both with epochs followed by the same label (periods of same vigilance state) or followed by a different label (transitions). Pairs containing the noise label were excluded from the depiction.

### 2.3 Analysis of scored data

To validate the existence of the defined states, a battery of advanced analysis was applied, as described below. This further serves as a framework for what can be expected from these analyses in various states.

#### 2.3.1 Coherence

Wavelet coherence describes the correlation of two signals. In neuroscience, coherence becomes a method for disentangling network dynamics, in line with C. Shatz’s paraphrase of the Hebbian paradigm ‘neurons that fire together wire together’ (Shatz, 1992). The electrode design used for this study yields six pairs of visual and auditory electrodes (Supplementary Figure S2). Wavelet coherence was computed with the built-in MATLAB function yielding the correlation coefficient. As the coherence was stable within each state (data not shown), the time dimension was averaged out. The resulting graphs are shown in Supplementary Figure S2.

When coherence was measured over the frequency spectrum from 0.5 to 70 Hz, a clear peak showed. The frequency of maximum coherence was then extracted from the graph. Both peak frequency and maximum coherence varied with state.

#### 2.3.2 Phase-amplitude coupling (PAC)

PAC describes the process where the phase of a low frequency predicts the amplitude of a higher frequency. PAC is computed by extracting the power of the high frequencies here 30, 35, 40, 45, and 50 Hz and the phase of the low frequencies 1–15 Hz. The PAC is the mean of the power multiplied by the *e* of the phase squared. The script is adapted from Cohen (2014), to fit data structures in the SAS pipeline. Since the phase variable is not very stable, then the phases were bootstrapped yielding the PACz instead of ‘just’ the PAC. The PACz is more stable across individuals and trials because it is normalized to 100 permutations of the signal.

The maximum PACz value was extracted including peak frequencies and then plotted in Supplementary Figures S3, S6, S7; in addition, the frequency of the lower frequency was used for statistics.

#### 2.3.3 Detrended fluctuation analysis and functional E/I balance

DFA was carried out to compute the α exponent of the filtered but not downsampled data. The script was adapted from Mike X. Cohen’s course on Udemy.^2^ The detrended fluctuation analysis was introduced by Peng et al. in 1994, to detect long-term correlations or power-law scaling of a signal (Peng et al., 1994). The power-law scaling has been linked to systems operating at criticality. A dynamic system is considered to be operating at an optimal level, when operating close to criticality, as this is the point at which shifting between two states is equally likely.

The α exponent is computed as the linear fit of the root mean square of the detrended epochs at 20 different scales, ranging from 1 to 20% of the epoch. The α exponents across the frequencies 0.5–70 Hz are shown in Supplementary Figure S4.

The crude meaning of the α exponent in relation to electrophysiology is as follows: α < 0.5, the process exhibits anti-correlations (unusual in EEG), α = 0.5; white noise, α = 1; the signal is made up of the same shape but possibly repeated on different scales i.e. the process has a memory (Hardstone et al., 2012). In addition, pink noise has an α ≈ 1. α > 1 suggests insufficient data for the computation.

Bruining et al. have described how the DFA can be used to generate a measure of the functional balance between inhibition and excitation (Bruining et al., 2020), by computing the correlation between the DFA and the original amplitude of the signal and subtracting it from 1. The fE/I is not computed if α < 0.6. The fE/I script from Bruining et al. was added to the DFA script. The resulting fE/I is shown in Supplementary Figure S4.

#### 2.3.4 Aperiodic component

The aperiodic component has gained interest as a potential biomarker for various psychiatry conditions (Voytek and Knight, 2015; Østergaard et al., 2024). The aperiodic component from the ‘fitting oscillations and one over f’ (FOOOF) analysis pipeline (available on Github)(Donoghue et al., 2020) is the slope of the 1/f. The 1/f signal is found in most natural signals including EEG. To fit the data into the FOOOF pipeline, the Welch’s power spectral density (PSD) estimation was applied to the scored signal and then exported to python, where the aperiodic component was extracted. The aperiodic exponent for each state is shown in Supplementary Figure S5.

#### 2.3.5 Spindle detection

Spindles were detected using a custom script with parameters from previous studies (Andrillon et al., 2011; Phillips et al., 2012). The signal was narrowband filtered in the range 8–18 Hz with a FIR filter; then, the z-scores were computed, and a Hilbert envelope of the z-scores was computed. From the envelope, spindles were identified as peaks with SD > 3.5 with duration of 0.5–2 s from the envelope increasing above SD = 1 to SD < 1. The average PSD from 9 to 16 Hz was then calculated for each sleep state (Supplementary Figure S6).

### 2.4 Statistics

Descriptive statistics are presented as mean and coefficient of variance (CV). The CV is the standard deviation divided by the mean, giving a coefficient that is comparable across data types.

Statistics were carried out in the R interface Rstudio (Rstudio, USA). Analyses of variance (ANOVAs) were computed of model variables (coherence, PACz, etc.) as functions of state with the ID as random effect, and all models showed a significant effect of state. *Post-hoc* tests were carried out using the estimation statistics package DABESTR (Ho et al., 2019).

The colormap of the correlogram was thresholded with the *p*-value adjusted for multiple comparisons, and a *t*-test (including F-statistics) of the linear fit was carried out to look at the correlation between states.

Statistics on curves were computed in MATLAB 2020a (MathWorks, USA), as asterisks when the Bonferroni corrected 95% confidence interval of the difference wave does not include zero.

## 3 Results

### 3.1 State definitions

**Figure 1:**
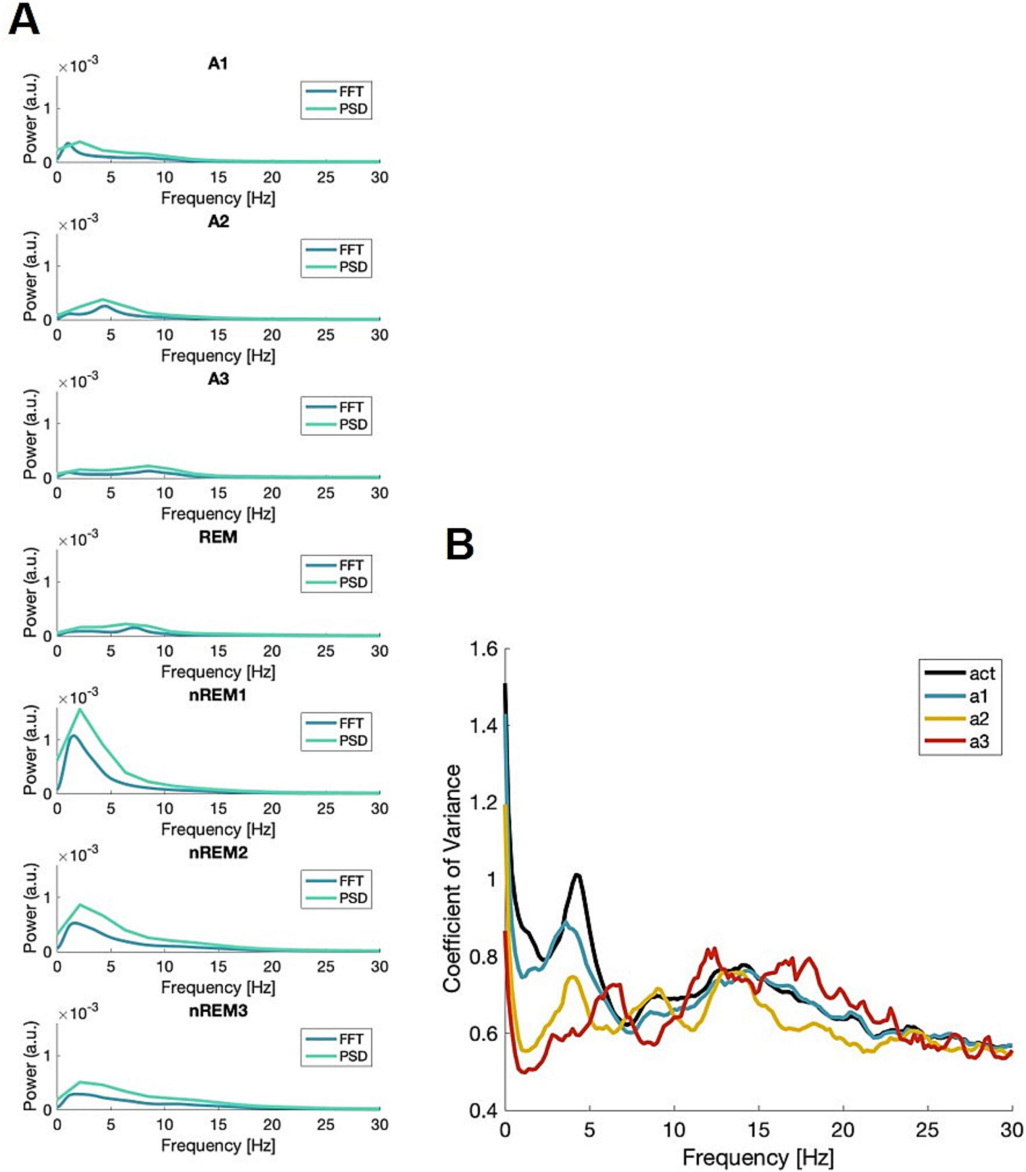
Frequency profiles of all states. **(A)** The profiles of all states used in scoring, both with the profile from FFT (AccuSleep) (blue) and the power spectral density using Welch’s (turquoise). **(B)** The coefficient of variance (CV) from the FFT, for the three awake states (a1; blue, a2; yellow, a3; red) along with the total awake (black). A.u.—arbitrary unit.

To validate the new scoring method, multiple datasets were employed. The data from Bl6 males and females is used for illustrations (Figure 1 and Table 1, including Supplementary Figures S2–S6), while data from Nrxn1 (Supplementary Figure S7) and Pcdh9 (Supplementary Figure S8) lines can be found in the Supplementary Materials. To visualize the power-frequency profiles, averages were made across epochs within each state. The averages are shown in Figure 1. The figure shows both the outcome of the fast Fourier transform (FFT) which is used in the present script, along with the power spectral density (PSD) which is used both in the FOOOF pipeline (Donoghue et al., 2020) and for sleep spindle analysis (Supplementary Figure S6). Both methods for computing power in the signal revealed the same peak frequency for each state. Panel B shows the coefficient of variance (CV) for the awake epochs when divided into substates vs. not divided, indicating that subdividing the awake epochs lowers variance in the data.

**Table 1:** Overview of states for the Bl6 (*n* = 19) with name, label, definition, mean frequency of maximum coherence (coherence is average for all five channel pairs) along with mean (CV), phase frequency of the maximum PACz value, and DFA; peak frequency and α exponent, and fE/I; frequency of maximum and minimum (Supplementary Figures S2–S4).

Name of state	ASCII label	Definitions for each epoch	Coherence Max. frequency Coherence value	PACz Max. phase frequency PACz mean	DFA Peak frequency, α exponent	fE/I Max. frequency Min. frequency
a1 (awake, active)	a (97)	Max power in the 0.2–3 Hz range	**8.8 Hz (0.06)**, 0.76 (0.14)	**6.9 Hz (0.59)**, 12 (0.59)	**P1: 5 Hz (0.16)**, α 0.86 (0.10) **P2: 10 Hz (0.14)**, α 0.84 (0.11)	**5.6 Hz (0.48)**, 0.91 (0.14) **14 Hz (0.26)**, 0.69 (0.30)
a2 (awake, not necessarily active)	b (98)	Max power in the 3.2–6 Hz range	**7.4 Hz (0.13)**, 0.69 (0.17)	**8.1 Hz (0.52)**, 5.3 (0.77)	**P1: 4.1 Hz (0.51)**, α 0.79 (0.16) **P2: 11 Hz (0.24)**, α 0.77 (0.09)	**4.2 Hz (0.29)**, 1.0 (0.10) **16 Hz (0.53)**, 0.63 (0.16)
a3 (resting state, inactive)	c (99)	Max power in the 6.2–12 Hz range	**8.8 Hz (0.07)**, 0.75 (0.15)	**7.3 Hz (0.55)**, 3.2 (1.19)	**P: 11 Hz (0.25)**, α 0.74 (0.13)	**5.1 Hz (1.47)**, 1.1 (0.27) **17 Hz (0.34)**, 0.27 (0.44)
REM (sleep)	l (108)	Power in the range 6–10 Hz > 0.2–6 Hz	**8.3 Hz (0.05)**, 0.82 (0.09)	**7.3 Hz (0.26)**, 10 (0.69)	**P1: 5 Hz (0.16)**, α 0.71 (0.09) **P2: 13 Hz (0.19)**, α 0.74 (0.10)	**6.9 Hz (1.01)**, 1.2 (0.09) **19 Hz (0.51)**, 0.62 (0.13)
nREM1 (slow-wave sleep)	m (109)	Power in the range 0.2–12 Hz more than 20 times the power in the range 20–24 Hz	**2 Hz (0.16)**, 0.81 (0.10)	**2.1 Hz (0.46)**, 27 (0.02)	**P: 1.6 Hz (1.06)**, α 0.70 (0.06)	**3.4 Hz (0.44)**, 1.2 (0.10) **15 Hz (0.07)**, 0.70 (0.19)
nREM2 (slightly faster than slow-wave)	n (110)	Power in the range 0.2–12 Hz 10–20 times the power in the range 20–24 Hz	**1.9 Hz (0.37)**, 0.73 (0.15)	**3.6 Hz (0.16)**, 17 (0.65)	**P: 1.5 Hz (0.66)**, α 0.77 (0.09)	**3.4 Hz (0.35)**, 1.1 (0.06) **16 Hz (0.26)**, 0.57 (0.11)
nREM3 (includes sleep spindles)	o (111)	Power in the range 0.2–12 Hz is less than 10 times the power in the range 20–24 Hz	**1.6 Hz (0.56)**, 0.64 (0.19)	**6.3 Hz (0.49)**, 8.8 (0.82)	**P1: 2.4 Hz (0.54)**, α 0.92 (0.08) **P2: 13 Hz (0.10)**, α 0.9 (0.07)	**3.0 Hz (0.6)**, 0.96 (0.09) **20 Hz (0.36)**, 0.45 (0.16)
Noise	N (85)	Power is too big or too low to fall into any of the other categories.				

The peak frequencies presented here are reproducible in the datasets with Pcdh9 and Nrxn1 (Supplementary Tables S2, S3). Bold writing in the table indicates the frequency of the maximum value with the CV in parenthesis.

As seen in Figure 1, each vigilance state has a unique frequency profile. To support this claim, a range of advanced analyses were done yielding: coherence, PACz, and α exponent (Supplementary Figures S2–S6). Subsequently, ANOVAs were computed to test for the effect of vigilance state. The resulting statistics were as follows: coherence *F*(6,612) = 98, *p* < 0.0001, PACz *F*(6,108) = 33, p < 0.0001, α *F*(6,234) = 50, p < 0.0001, fE/I *F*(6,194) = 20, p < 0.0001, and the aperiodic component F(6,108) = 554, *p* < 0.0001. The *post-hoc* analysis is summarized in Supplementary Table S1. In conclusion, the effect of the vigilance state was highly significant for the measures used here. Table 1 displays the average maximum frequency and related measure with CV in parenthesis. The aperiodic exponent describes an underlying activity and changes with state, over the frequency range 0.5–65 Hz (Supplementary Figure S5).

There were recurrent trends in the frequency profiles of the vigilance states (Table 1). The active states share the high peak frequency with REM sleep. The sleep states share the low peak frequency. In the active states, it was noticeable that the frequencies of maximum coherence and PACz were in the 6.9–8.8 Hz range when the definitions of a1 and a2 have peak frequencies below 6.2 Hz. REM sleep shared several features with the awake states and looked similar to a3 when considering the peak frequencies along with α, fE/I, and aperiodic exponent (Supplementary Table S1) but not when looking at the coherence or PACz, i.e., REM showed higher coherence and higher PACz compared to a3.

The DFA showed that the point of highest criticality varied with frequency and vigilance state, which could suggest that the frequency bands have a practical meaning for the functioning. The analysis of the functional excitation-inhibition balance (fE/I) showed one peak over 1 (excitation-dominated), followed by a peak below 1 (inhibition-dominated). It can be speculated that this might be a way for the system to isolate frequencies if different frequencies have different interpretations or network properties. Interestingly, the beta band (12–30 Hz) is inhibition-dominated in all states, yet spindle analysis shows activity in the lower beta band (<13 Hz) during nREM2 and nREM3 (Supplementary Figure S6S1). Spindles are detectable in the frontal channel in nREM2 and nREM3, with a peak frequency at 11.2 Hz. The PSD of the frontal channel changes across sleep states, while the PSDs in the auditory and visual channels stay at the same level.

These patterns were robust across datasets and time (Supplementary Figures S7, S8 and Supplementary Tables S2, S3). It is noticeable that, although the PACz values for a2 and a3 are low, then they center around the same frequencies in all datasets: Bl6 (Table 1), Nrxn1 (Supplementary Table S2), and Pcdh9 (Supplementary Table S3), indicating that these features are robust, in spite of the differing sources of variation in the datasets: The Bl6 data contain both males and females, while the Nrxn1 and Pcdh9 data contain multiple genotypes recorded at different time points.

#### 3.1.1 Comparison of SAS and manually scoring

**Figure 2:**
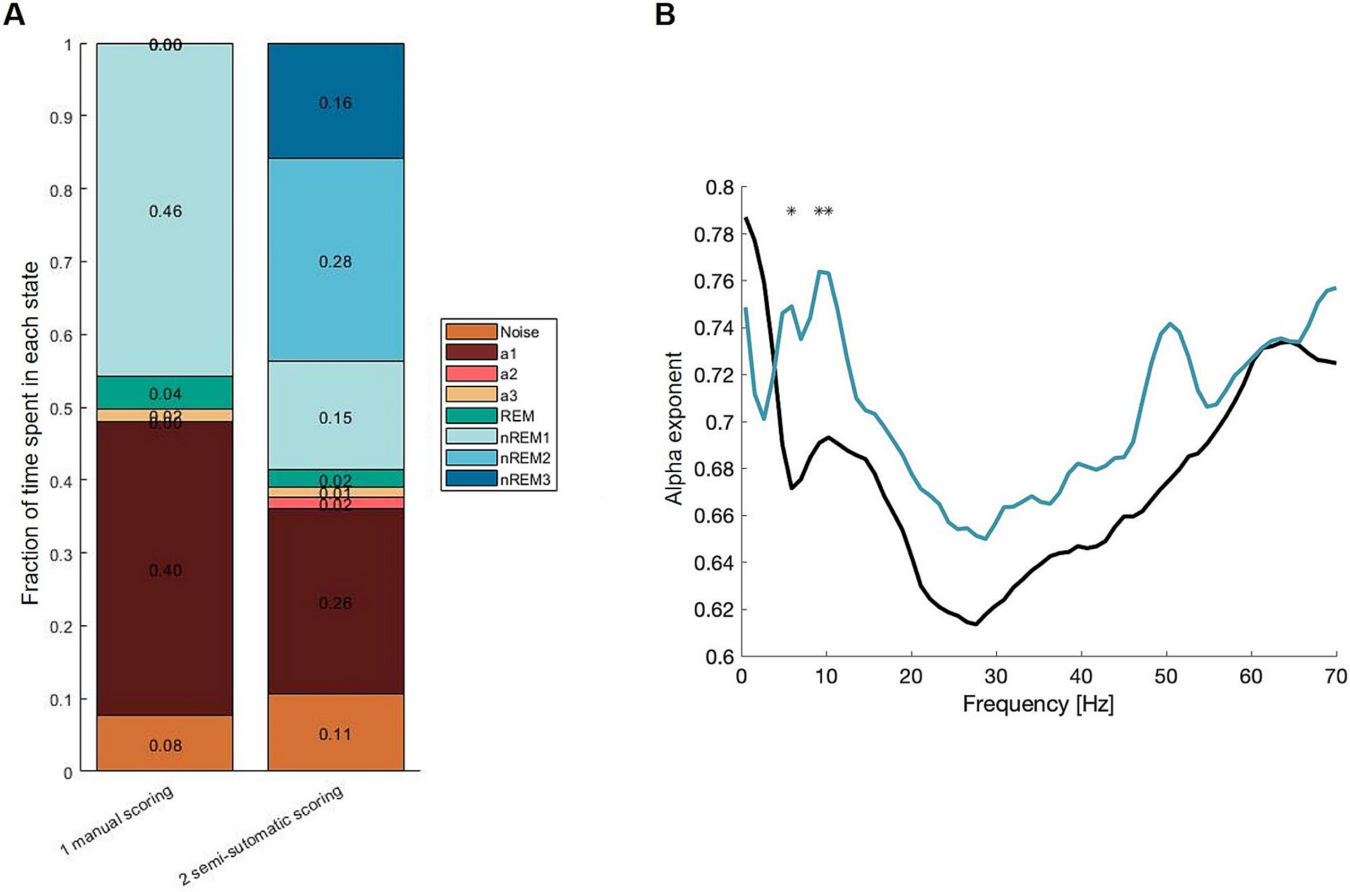
**(A)** Summary of labels from manual scoring and semi-automatic scoring. **(B)** Mean α exponent. Black: manual scoring, turquois: SAS. The two curves differ significantly at three frequencies out of 65. This happens in Asterisk where the CI95 (Bonferroni-adjusted) of the difference wave does not include 0. This happens at the alpha band frequency.

Data from *FMR1* knockout mice were used to compare semi-automatic scoring with the current standard which is manual scoring by two people. It should be noted that the FMR1 data were scored with a focus on the resting state (a3). Figure 2A shows a bar plot of vigilance states, when manually scored vs. semi-automatically scored. The histogram has been organized to give an idea of how the entire dataset looks.

The fraction of resting state is ~2% in the manually scored data and ~1% for the same data scored semi-automatically.

To test the consequences of the different scoring methods, secondary outcomes were used. Here, *α* computed with DFA is used to compare the two methods (Figure 2B). SAS shows a significantly higher α exponent at the alpha-band ~10 Hz. Overall, the shapes are similar, and only 3 out of 65 frequencies are significantly different (illustrated with asterisks). The α exponents for the SAS data are higher at 7 and 10 Hz, indicating the SAS is more accurate, where the manually scored epochs may contain different vigilance states giving the signal a more random appearance—lowering the α exponent.

#### 3.1.2 Epoch length does not influence the α exponent

**Figure 3:**
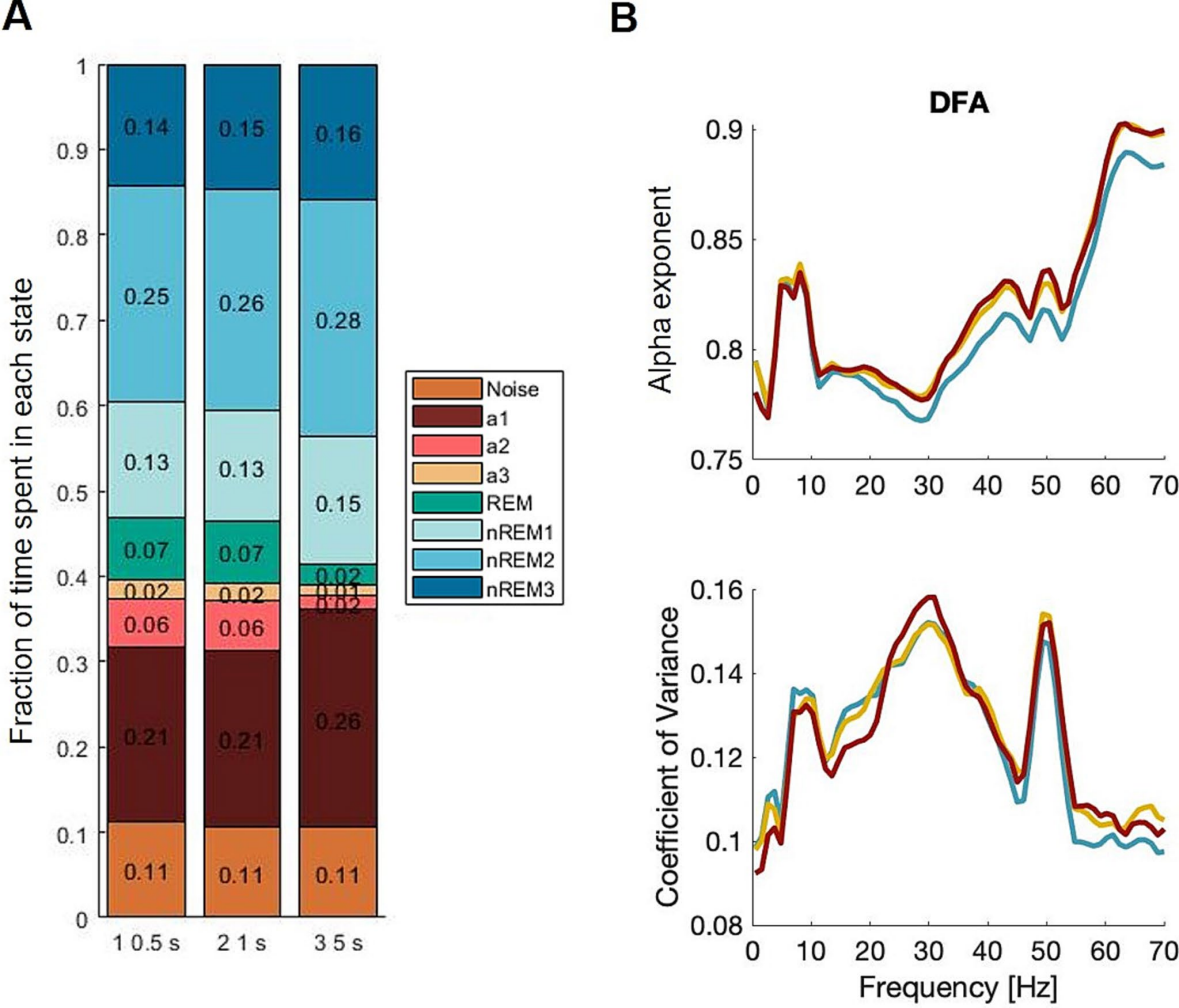
Epoch lengths primarily showed an effect on the less frequent states, such as theta dominated awake, alpha dominated awake (resting state), and REM sleep. To have comparable amounts of data, we used the α exponent of the total awake state data and not the three substates separately. Blue; 0.5 s, yellow; 1 s, red; 5 s.

The manual scoring (and SAS comparison) was carried out with 5 s epochs. To test how the α exponent was affected by the length of the epochs and essentially the amount of data in each epoch, semi-automatic scoring was carried out with epochs of 0.5, 1, and 5 s (Figure 3). Since the longer epochs would be expected to contain more noise, then the α exponent would be expected to be generally lower for epochs of 5 s than epochs of 1 s which again would be lower for epochs of 0.5 s duration. Furthermore, it was assumed that there was a slight variation in the heterogeneity of data as the vigilance states seem to change rapidly, and the 5 s epochs may contain a greater mix of states than the 1 s epochs. However, the results showed a minimum of difference in α exponents, and the CVs are the same across epoch lengths.

To summarize, the epoch length mattered for the amount of data in each vigilance state but did not introduce significant changes in advanced outcomes such as the α exponent.

### 3.2 24-h recording of Nrxn1-deficient mice

**Figure 4:**
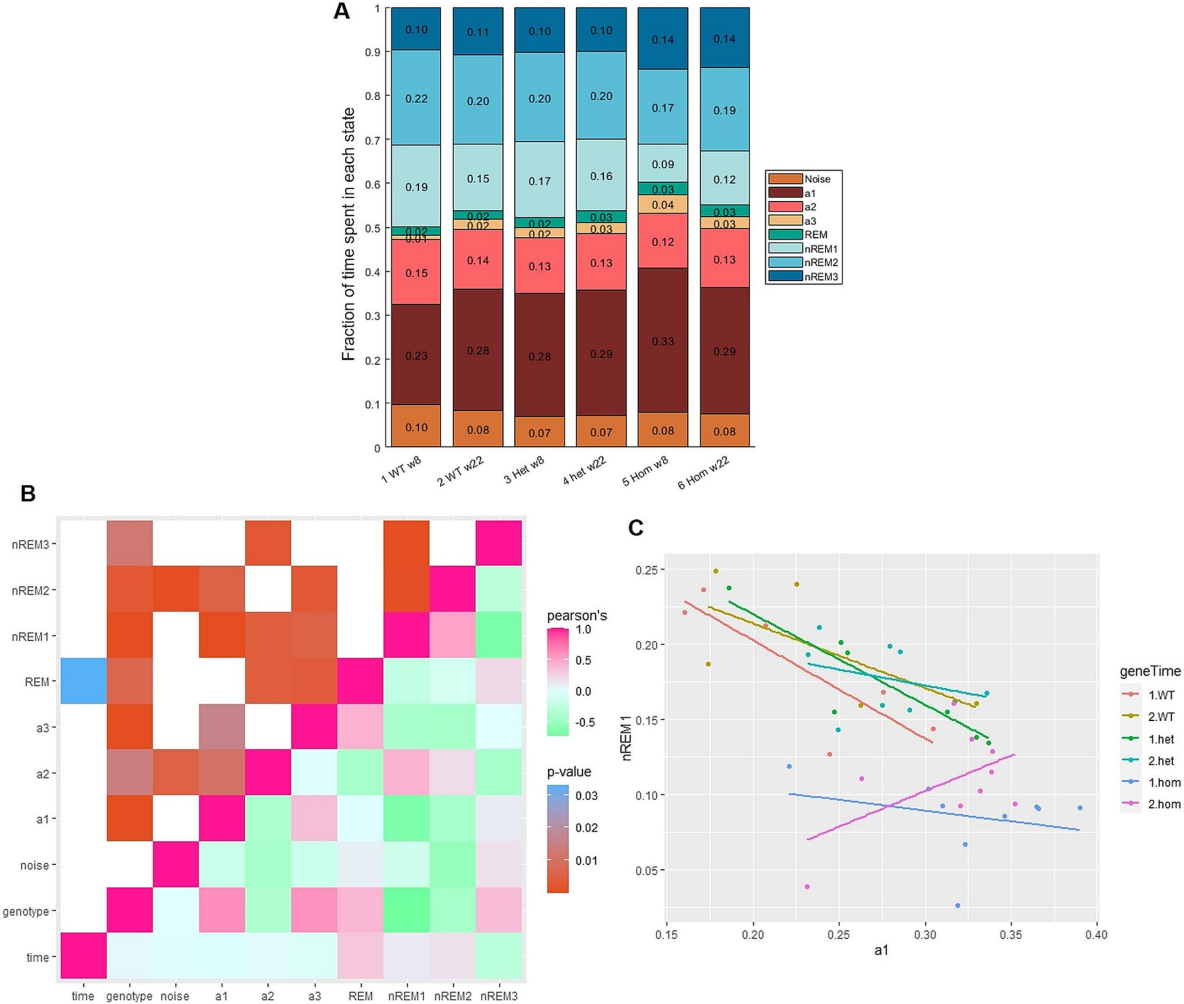
Proportion of vigilance states varies with genotype. **(A)** Summary of label distribution for three genotypes [wild-type (WT), heterozygous (Het), and homozygous (Hom)] at two time points [8 weeks (w8) and 22 weeks (w22) of age] scored with 1 s epochs. **(B)** Correlation plot of variables: rose is Pearson’s correlation coefficient of 1 = positive correlation, green: ~ − 1 = negative correlation. Mint: 0, no relation between variables. Age showed barely any correlation but a significant relation to REM (*p* = 0.03). Genotype showed statistically significant relations to all states especially nREM1 (Pearson’s = −0.74, *p* < 0.0001). **(C)** Correlation of proportion of nREM1 and a1, slope = −1.1 for WT and Het, but not for Hom at the second time point slope = 0.64.

The semi-automatic scoring was applied to investigate the sleep and awake distributions in the Nrxn1-deficient mouse model (both homozygous and heterozygous knock-out mice) and their wild-type littermate controls. Recordings were made over 24 h when the animals were 8 weeks old and again at 22 weeks. The summary data showed that sleep/wake distribution changed as a function of genotype (Figure 4A). In fact, genotype correlated significantly with all states (Figure 4B). Homozygous knock-out (Hom) mice showed significant increases (mean [95% confidence interval]) in the number of epochs labeled: a1 (Tp1: 9.95% [4.46 14.8], Tp2: 8.6% [3.23 13.1]), a3 (Tp1: 3.12% [2.29 4.07], Tp2: 2.44% [1.32 4.2]), and REM (Tp1: 0.86% [0.11 1.65], Tp2: 1.41% [0.69 2.24]), and a decrease in epochs labeled nREM1 (Tp1: −9.96% [−13.7–6.31], Tp2: −7.62% [−11.5–3.79]) at both time points when compared to WT. Fewer epochs were labeled nREM2 (−4.44% [−7.51–1.49]), while the number of epochs labeled nREM3 (4.34% [0.68 10.4]) was increased at week 8 only. Heterozygous knock-out (Het) mice showed significant increases in epochs labeled as a3 (Tp1: 1.24% [0.05 2.62], Tp2: 1.41% [0.17 2.81]) and REM (Tp2: 1.09% [0.31 1.83]).

The correlogram (Figure 4B) showed the strongest correlation (with high Pearson’s and low *p*-value) between a1 and nREM1. The correlation between nREM1 and a1, which is ~ − 1 (Figure 4C), in other words each minute not spent asleep (slow wave), is spent awake (and active) and vice versa. This is not the case for the Hom group at 22 weeks of age, where there is a weakly positive correlation between a1 and nREM1. nREM3 showed a statistically significant, negative correlation with nREM1 (Figure 4B). As state distribution is a zero-sum game, then it is likely that the increases in nREM3 are resulting from the imbalance between a1 and nREM1.

**Figure 5:**
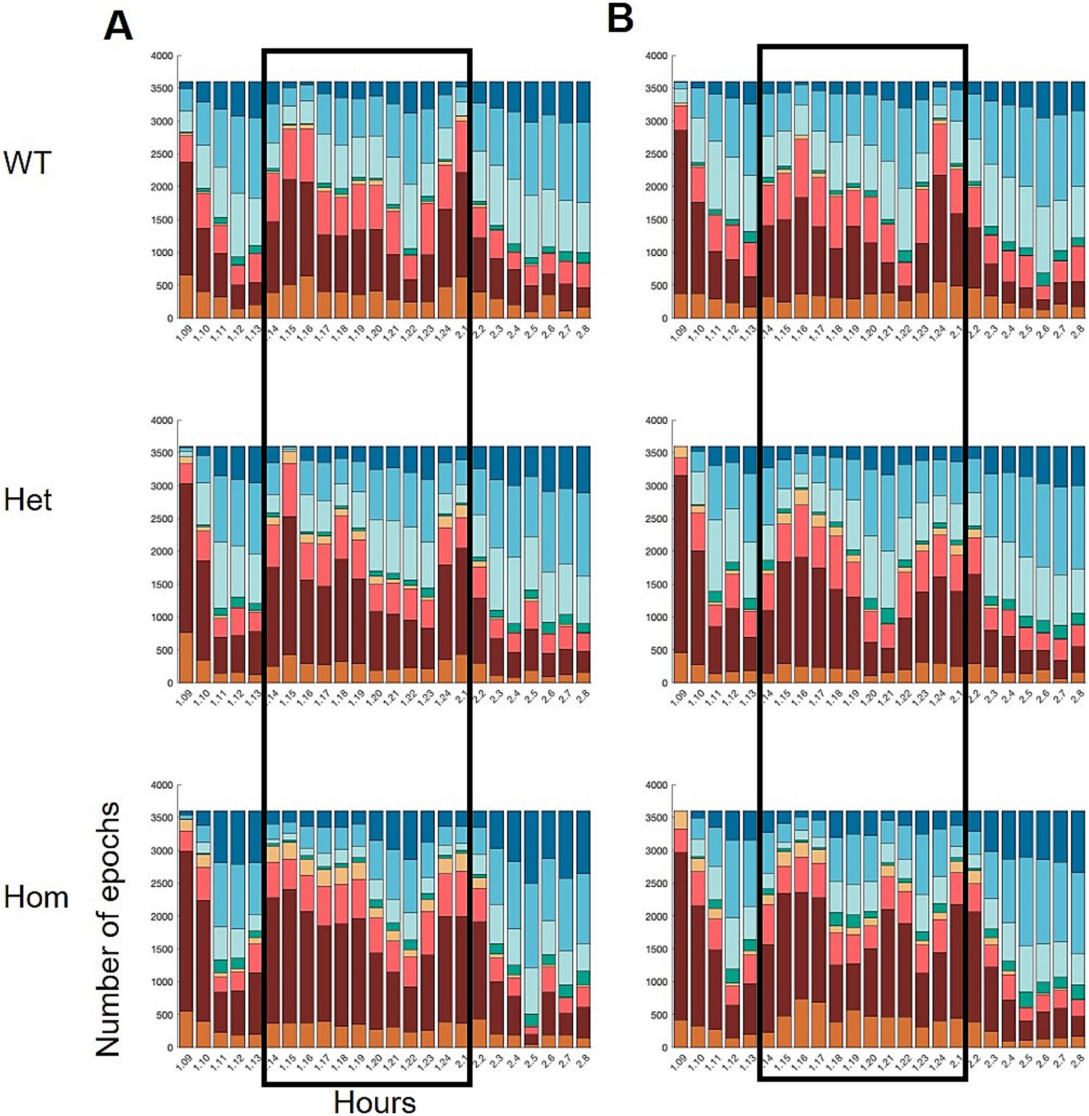
Hourly distribution of wake and sleep states over the course of 24 h, starting at 9 am on day 1 and ending at 9 am on day 2 (x-axis). The dark phase is indicated by the black frames. Wild-type (WT), heterozygous (Het), and homozygous KO (Hom) of Nrxn1 at **(A)** 8 weeks (young adult) and **(B)** 22 weeks of age (middle-aged). Statistical analysis of the development in a1 and nREM1 epochs is shown in Figure 6.

Genotype effects on the sleep–wake distribution were also observed. Distributing the epoch labels over the course of 24 h showed that the Hom group changed their pattern of sleep–wake over time (Figure 5); at the second time point, the group has two increases in sleep during the active phase, instead of one.

**Figure 6:**
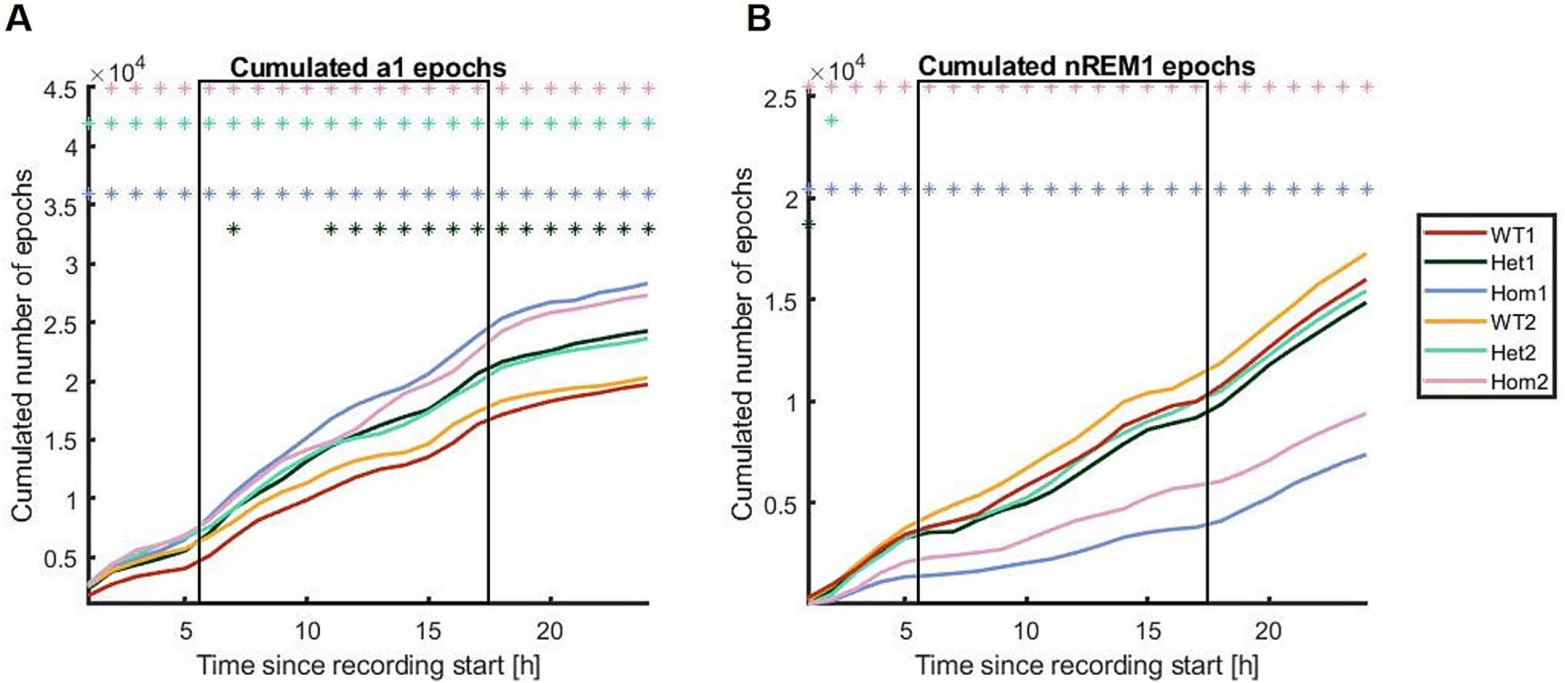
Cumulative distributions shown for **(A)** a1 and **(B)** nREM1 in all three genotypes studied at 8 and 22 weeks of age. The Hom group has both more a1 and less nREM1 than the other groups. The x-axes are the cumulated number of epochs; the y-axes are the proportion of data points (one step per hour). The square frames indicate the dark/active phase. The asterisks indicate when the Bonferroni-corrected CI95 of the difference curve to WT1 does not include 0, meaning a statistically significant difference. Red; WT time point 1 (8 weeks), dark green; Het 1, light blue; Hom 1, yellow; WT time point 2 (22 weeks), light green; Het 2, pink; Hom 2.

To scrutinize the distribution of sleep and wake over the 24 h period further, cumulated distributions were drawn using the epoch labels per hour (Figure 6). The cumulative plots suggested that the change in correlation is driven by changes in time spent in nREM1 rather than in a1, although Hom has more consecutive periods of awake. The Hom group showed less nREM1 and it is distributed differently over the course of the 24 h recordings, as the Hom group has a slow incline in sleep epochs during the active phase, where the wild-type and Het group has uneven and steeper slopes. Although the 24-h recording was carried out in the home cage, there was still a visible habituation period from getting the transmitter on (Figure 5).

When looking at the sequence data and transitions (Supplementary Figure S10), then the early time point of the Hom group (8-week-old animals) stands out, with more transitions between awake states and fewer consecutive nREM1 periods (Supplementary Figure S11). The later time point (22-week-old animals) seemed to be closer to the WT and Het groups; this is contrary to Figures 4C and 5 where the later time point differs from the rest. These findings suggest that the Hom group is employing some alternative compensation strategies to cope with the lack of slow-wave sleep, and these strategies are only working in part.

In summary, there is disorder in the sleep–wake balance of the homozygous knock-out generally expressed as less slow-wave sleep. Furthermore, the changes in sleep distribution in the Hom group did not fully compensate for the underlying lack of nREM1.

## 4 Discussion

In this study, we presented a guideline and tool for scoring electrophysiological data into vigilance states and provided concrete examples of how vigilance states matter for advanced outcomes (i.e., coherence, PACz, α, fE/I, and aperiodic exponent). The new method for scoring electrophysiological data used fixed frequency profiles of each state. We decided to keep the frequency bands defining each state fixed as they reoccur with the same ranges. In addition, the assignment was based on the summed power in a range rather than the peak frequency as we believe the majority of the power should determine the assignment rather than the power at a specific peak frequency. The definitions are exclusive to each vigilance state but also written in such a way that *all* epochs are assigned an appropriate label (including noise). With the current version, a 24-h recording can be fully scored in approximately 1 h, which includes quality checks. Furthermore, we showed how advanced outcomes were stable across datasets.

This SAS method should be applied to EEG data in general as recent research has shown that scoring even awake data from humans into vigilance states can determine whether you find a difference between groups or not (Østergaard et al., 2024).

### 4.1 Number of states

Traditionally, scoring of EEG data has only been applied to sleep data, while the awake substates have not been studied in depth as a lot of animal electrophysiology has been carried out in anaesthetized animals (Vassalli and Franken, 2017) and human subjects have mostly been sitting still. Here, the purpose was to ensure that all data were scored and that every frequency profile of each state was represented fairly in the definitions. This resulted in seven distinct vigilance states, namely, three awake states and four sleep states. During nREM sleep, delta power occurs as a spectrum starting with high power at low frequency that gradually decreases while ‘spreading’ over more frequencies to start over again; this cycle is occasionally interrupted by REM sleep. The golden standard of sleep scoring is ‘the AASM manual for scoring of sleep and associated events’ which is developed for humans. In this manual, not all states are translational because they involve eye movement, chin EMG, or specific voltage such as N3 where ‘a peak-to-peak amplitude of >75 μV’ is expected. One should generally be careful translating voltages between cortical and extracranial electrodes. Comparing the state definitions of the present study to the AASM manual (Troester et al., 2023), nREM1 shows high amplitudes and slow wave activity resembling AASMs N3, while nREM2 and nREM3 have sleep spindles similar to N2. In the present definition where the relative power within the epoch determines the label, a gradient resulting from the spindles is not surprising. The N1 definition is not used here as the wake–sleep transitions in mice are usually from wake to slow-wave delta sleep and not to a state of ‘low-amplitude mixed frequencies in the theta range 4–7 Hz’. The transition analysis of the SAS (Supplementary Figure S10B) shows a lot of fluctuation between the sleep states. Some of these transitions may be true, but some might also be caused by placing a hard boundary in a spectrum. This scoring does not take epileptiform activity into account. That said most seizure activity would ‘distort’ the frequency power spectra by increasing power in the higher frequencies (>13 Hz); therefore, more a3 and nREM3 would be expected.

#### 4.1.1 Spectral fingerprint of state

Each state (both wake and sleep) is dominated by a frequency profile with one prominent frequency band. These prominent frequency bands reoccur to some extend in the advanced measures, e.g., coherence and PACz. They may be related to a default network mode. Neurons cannot be inactive, so either they are in a default mode (not to be confused with the default mode network) or active—processing stimuli. This would explain why the power of the awake states is generally lower than for the sleep states; there are fewer neurons in the default mode as there is a lot of ongoing and specific sensory processing. In relation to human data, then it is well-established that the alpha power increases over the occipital lobe when the eyes are closed (Travis et al., 1974); less input means more neurons idling (Kropotov, 2016) or in the default mode. This default mode may be the state in which the neurons are the readiest for certain types of activity.

The delta band activity seen during a1 may be the default frequency in deeper structures. However, Schultheiss et al. studied electrophysiological activity of the hippocampus and described how delta-dominated network modes appear interspersed with theta-dominated modes during navigation (Schultheiss et al., 2020), thus representing distinct circuit dynamics. The neurons’ ability to fire on different frequencies may depend on anatomical location and connections as alpha band activity has not been reported for deeper structures. Theta activity is expected to originate in the hippocampus both during awake and during sleep (Vassalli and Franken, 2017). It is noticeable that the a2 shows a lower theta peak frequency than REM. More research is needed to determine whether this difference is related to the lower muscle tone during REM.

In one of Steriade’s last papers, he argued that instead of looking at single frequency bands, one should look at the combination of multiple bands as ‘complex wave sequences’. In the article, he described how oscillations in the beta band can move into the gamma band ‘under light membrane depolarization’ (Steriade, 2006). He argues that change in a single band is less important than the broader trend of electrical activity. The proposed model is not incompatible with the assumptions of SAS, and the model presented in the current paper focuses on activity in one band for labeling purposes only, while advanced analysis uses higher frequency resolutions <1 Hz.

### 4.2 Slow-wave sleep is decreased in Nrxn1α deficient mice

To provide an example of what can be gained from applying the pipeline to a genetic model of autism spectrum disorder, 24-h EEG recordings were analyzed. The 24-h recordings of the Nrxn1 model show clear correlations between the genotype and state (Figure 4B). In addition, the distribution of these states changes with time and genotype, aligning with the fact that sleep disturbances are often reported for autistic people (Devnani and Hegde, 2015). The results indicated that changes in a1 were dependent on the gene dosage, meaning that the Hom group had more epochs labeled a1 than Het, which had more than WT. The number of epochs labeled nREM1 was only affected in the Hom and not in the other groups (Figure 6). It can be speculated that there are more compensatory mechanism securing nREM1 than awake substates as slow-wave sleep is considered important for much of the maintenance of the brain (Léger et al., 2018). It has been shown that during slow-wave sleep, the interstitial space of the brain expands to make room for the glymphatic flow (Jessen et al., 2015) and a functional decoupling of the neurons is occurring (Steriade, 2006), as the liquid flow around the neurons is increasing. The massive increase in delta power seen during nREM1 may be caused by either decoupling of neurons, silencing the cortical neurons, and making the delta oscillation from deeper structures easier to record, or the delta band activity may be the base frequency of uncoupled neurons; thus, uncoupling causes cortical neurons to change from alpha to delta oscillatory activity. The cyclic sleep (Supplementary Figure S1) may reflect the system decoupling to clean the synapses and then gradually recoupling the networks again, to allow sensing of the environment, as functional coupling of neurons into networks is necessary for optimal functioning (Phillips et al., 2012; Kopell et al., 2014). Nrxn1α is a structural protein involved in the assembly of presynaptic proteins (Dean et al., 2003); thus, deficiency of the protein may cause a disturbance in the cortical neurons’ ability to modulate activity pattern. The increase in awake states and irritability (Etherton et al., 2009) may be a consequence of the disturbed sleep, thereby an indirect consequence of knocking-out Nrxn1α rather than a direct consequence of the knock-out.

Interestingly, not all awake states increased the same, while there were increases in a1 and a3 there was a decrease in a2 epochs. If Fiebelkorn and Kastner’s theory about theta-band activity being necessary for attention (Fiebelkorn and Kastner, 2019) is correct, then Nrxn1α-deficiency may cause a progressive decrease in attention and an increase in wakefulness.

### 4.3 Limitations

Although the scoring is mostly automatic, manual intervention may be needed in cases where noise or dropouts in the signal can make it hard for the change point analysis to locate ‘true’ sleep–wake transitions in the signal. Still, the overall processing time of the proposed method would be faster than fully hand scoring the data as the manual labor is only in indicating wake–sleep transitions and not in labeling every epoch.

It is a possible limitation that vigilance states may change within a fraction of a second, which was the current length of our epochs for the analysis. In such a situation, some epochs may contain half of one state and half of another state, increasing the variability within a state. Nevertheless, based on our analyses, an epoch length of half a second seemed to show similar distributions as an epoch length of 1 s (Figure 3), suggesting that states of less than 1-s duration are unlikely.

## 5 Conclusion

The semiautomatic scoring (SAS) tool presented here can identify seven distinct vigilance states. The main advantages of SAS are the speed of data processing and the robustness across datasets. Compared to manually scored data, the α exponents were very similar (Figure 2B), validating that the SAS tool is just as accurate as manual scoring. Furthermore, applying the SAS tool to a mouse model for synaptic dysfunction in ASD revealed a significant gene dosage effect in the number of a1 epochs. Thus, this new methodology puts forward an optimized and validated EEG analysis pipeline for the identification of translational electrophysiological biomarkers for brain disorders that are related to sleep architecture and E/I balance.

## Data Availability

The datasets presented in this study can be found in online repositories. The names of the repository/repositories and accession number(s) can be found in the article/Supplementary material.

## Footnotes

^1^ https://github.com/karthik/wesanderson/tree/master

^2^ https://www.udemy.com/course/solved-challenges-ants/
