# Supplementary material for: Seven unique frequency profiles for scoring vigilance states in preclinical electrophysiological data
Source: Front Neurosci. 2025 Apr 30;19:1488709. doi: 10.3389/fnins.2025.1488709 (PMC12075235; doi:10.3389/fnins.2025.1488709)
Supplement: Supplementary file 1 [file Data_Sheet_1.docx]

Supplementary Material - Seven unique frequency profiles for scoring vigilance states in preclinical electrophysiological data

# Canonical diagram


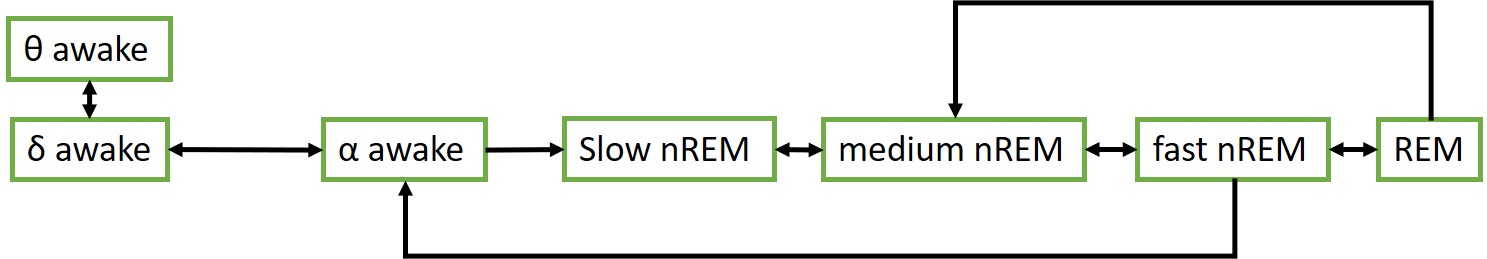


**Figure S1** Canonical diagram of transitions between states.

# Bl6 19 animals (9 male, 10 female) (8 weeks at the beginning)


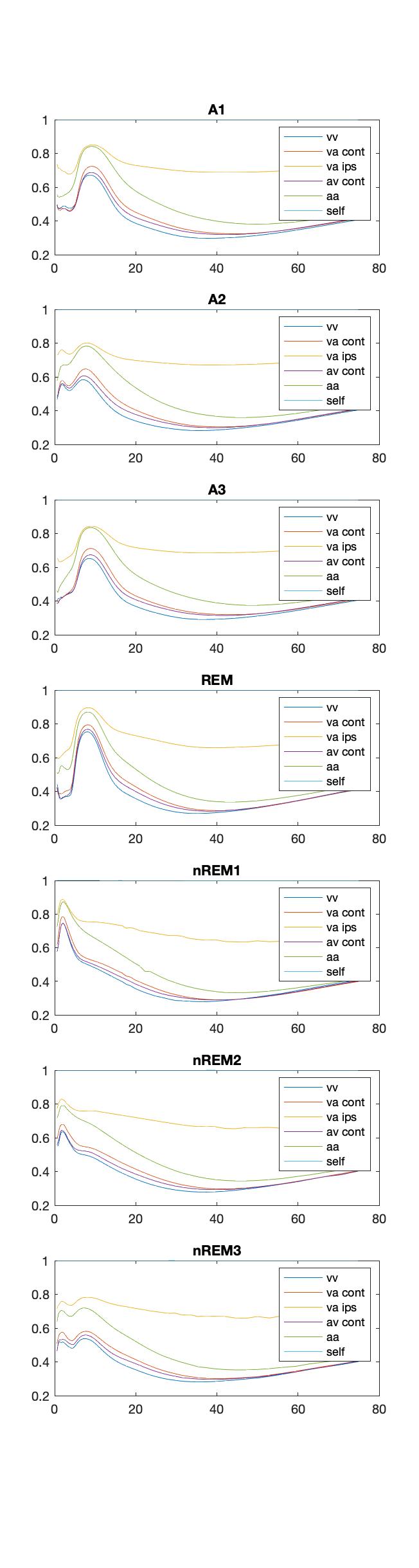

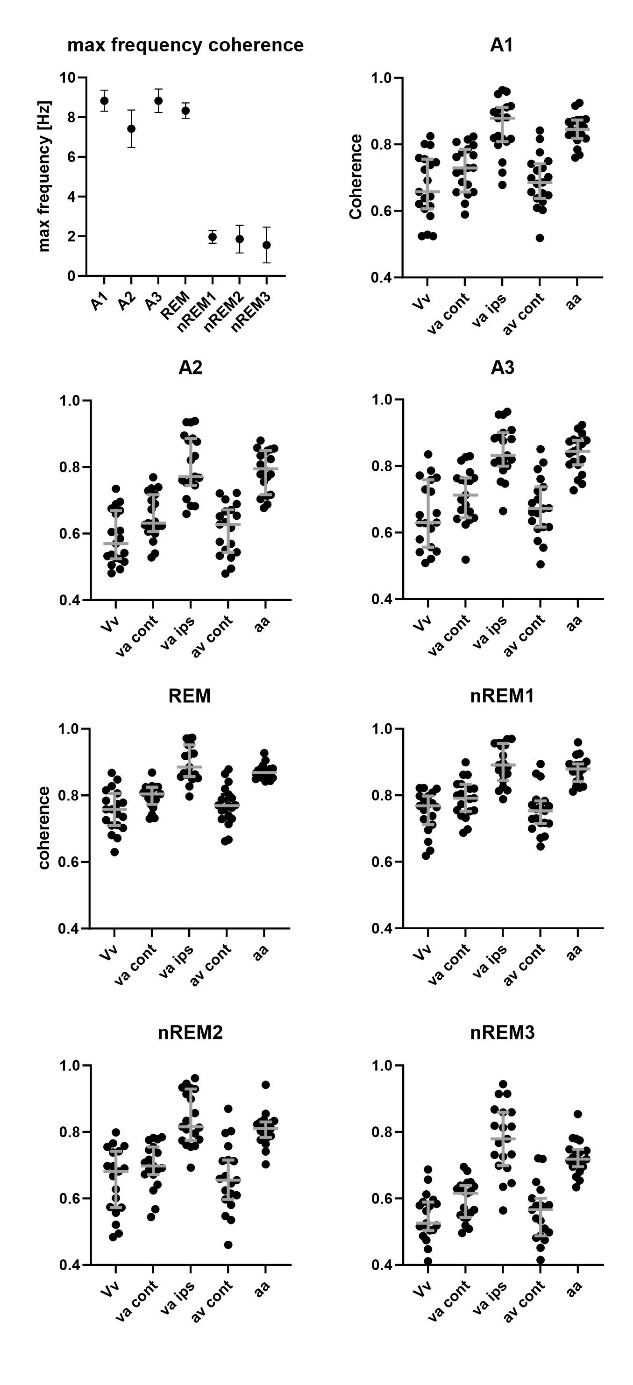

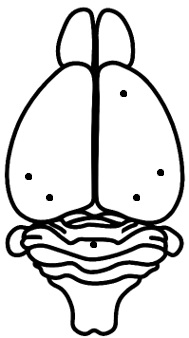


Aud

Vis

**A**

**B**

**C**

**Figure S2** Coherence. Left panels show coherence across frequencies for different states for blue: visual-visual (interhemispheric), red: visual-auditory (interhemispheric), yellow: visual-auditory (same hemisphere), purple: auditory-visual (interhemispheric), green: auditory-auditory (interhemispheric) and light blue: same electrode (constant coherence of 1). The electrode pairs are shown in diagram. The right panel shows the characteristics of the peaks from the left panels.


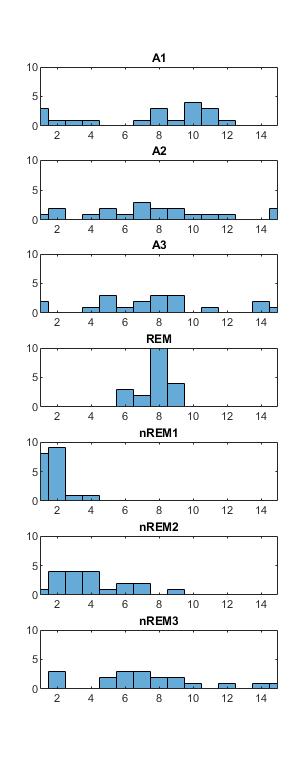

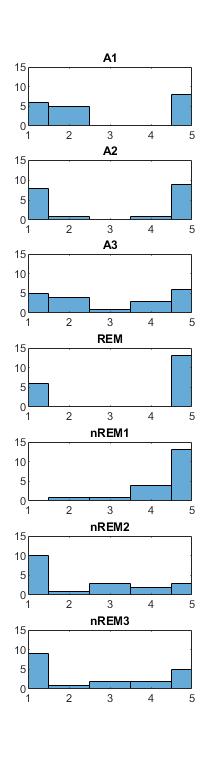

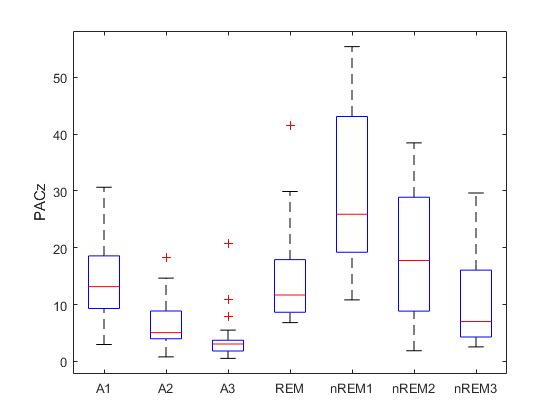


**A**

**B**

**C**

**Figure S3** The maximum PACz value, the lower frequencies (1-15 Hz), the higher freq. (30, 35, 40, 45, 55). Max. PACz value as boxplots for each state.


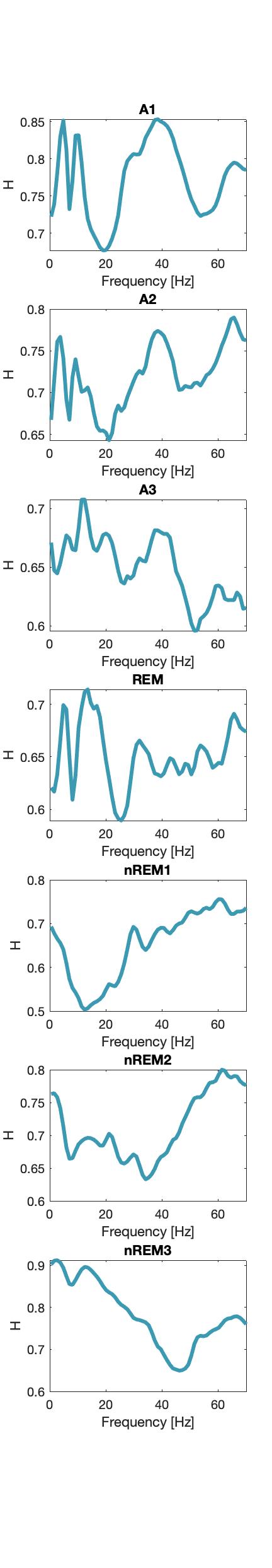

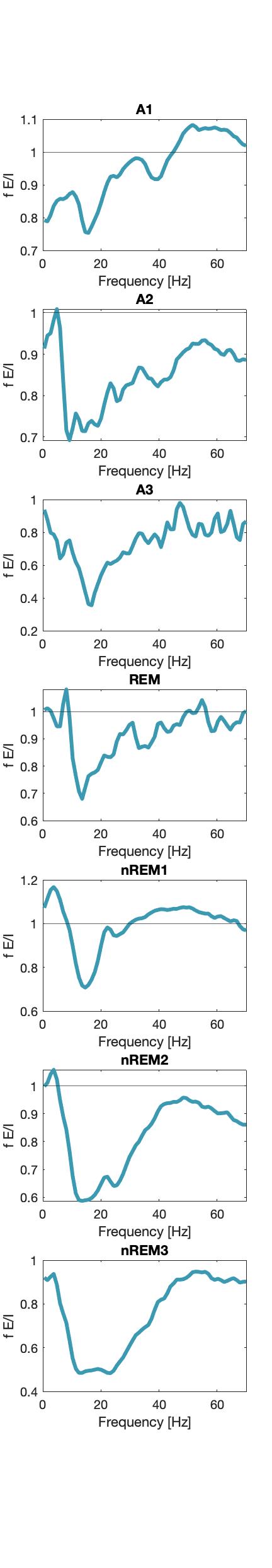


α

α

α

α

α

α

α

**Figure S4** Average DFA and fE/I from each state of the Bl6s, both male and female.


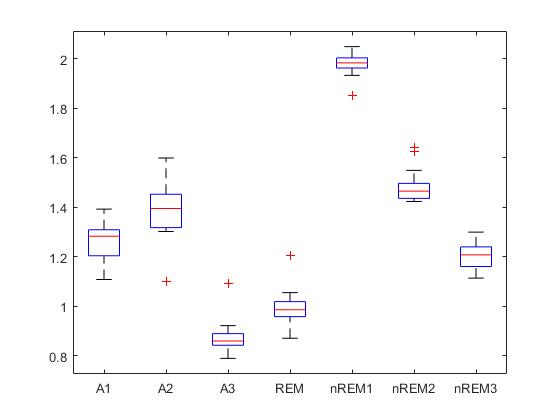


**Figure S5** Aperiodic exponent (from FOOOF) for each vigilance state, from all Bl6 animals. Red crosses indicate outliers.

**Table S1** Estimation statistics as post-hoc of the ANOVAs. States are compared to the a1 state. Displayed with mean and 95 confidence interval (CI95), the numbers in bold highlight cases where the CI95 doesn’t include zero.

| state | coherence | PACz | DFA | fE/I | Aperiodic exponent |
| --- | --- | --- | --- | --- | --- |
| A2 | **-0.0678**  **[-0.101; -0.0352]** | **-6.94**  **[-10.6; -3.32]** | **-0.0727**  **[-0.117; -0.0297]** | 0.0337  [-0.0677; 0.134] | **0.125**  **[0.0626; 0.182]** |
| A3 | -0.0113  [-0.043; 0.0198] | **-9.07**  **[-12.6; -5.48]** | **-0.126**  **[-0.168; -0.0817]** | -0.134  [-0.327; 0.075] | **-0.399**  **[-0.437; -0.352]** |
| REM | **0.0612**  **[0.0355; 0.0871]** | -2.32  [-6.2; 2.61] | **-0.129**  **[-0.169; -0.0948]** | 0.0901  [ -0.0424; 0.226] | **-0.277**  **[-0.318; -0.229]** |
| nREM1 | **0.0567**  **[0.0291; 0.0838]** | **14.5**  **[8.55; 21]** | **-0.21**  **[-0.25; -0.174]** | **0.146**  **[0.0325; 0.254]** | **0.711**  **[0.673; 0.749]** |
| nREM2 | -0.0238  [-0.0553; 0.00737] | 5.12  [-0.219; 10.7] | **-0.114**  **[-0.157; -0.079]** | 0.015  [-0.0966; 0.116] | **0.213**  **[0.174; 0.261]** |
| nREM3 | **-0.116**  **[-0.148; -0.0836]** | -3.49  [-7.69; 1.12] | **0.0567**  **[0.0156; 0.0903]** | -0.0979  [-0.211; 0.00595] | **-0.0633**  **[-0.101; -0.0231]** |


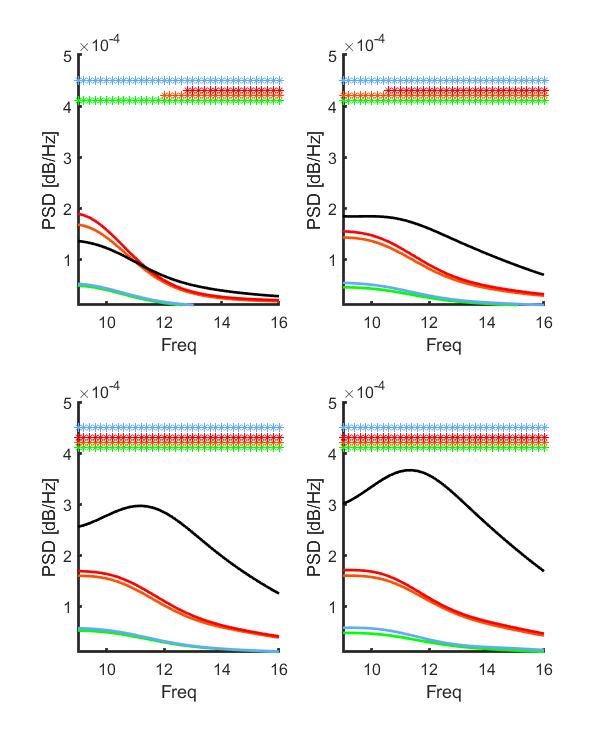


**REM**

**nREM1**

**nREM2**

**nREM3**

**Figure S6** Power spectral density (PSD) of sleep spindles during top: REM, nREM1; bottom: nREM2, nREM3. Black; frontal channel, green/blue; visual channels, orange/red; auditory channels. Asterisks indicate when the Bonferroni corrected confidence interval of the difference curve does not include zero.

# Nrxn1α 41-40 animals (all males), three genotypes, two time points (10 weeks, 22 weeks)


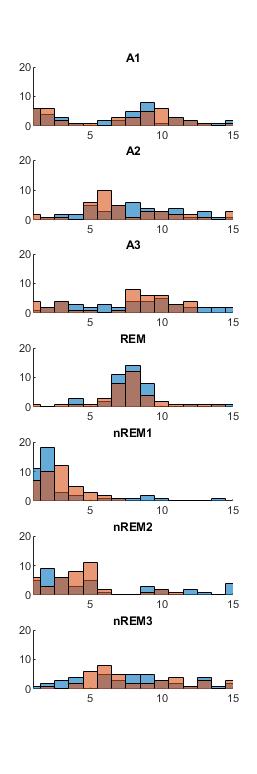

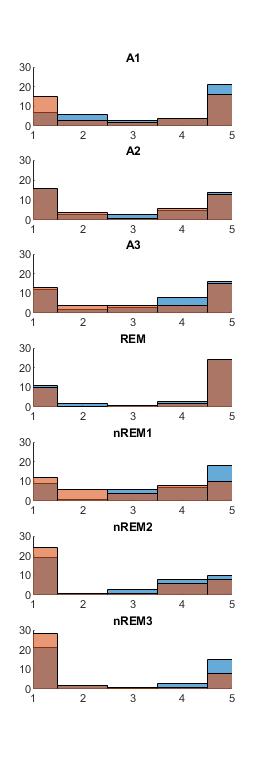

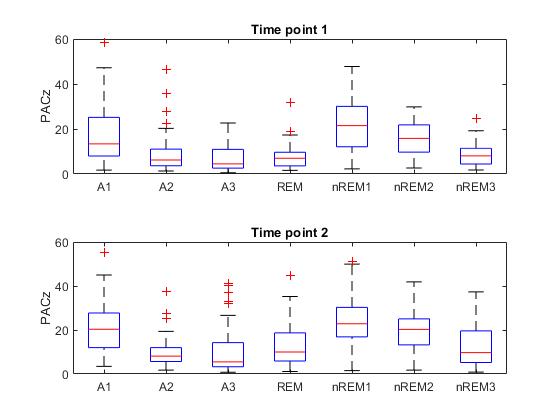


**A**

**B**

**C**

**Figure S7** Distributions of maximum PACz values of all genotypes, blue: 10 weeks of age, red are 22 weeks of age. All genotypes are pooled.

**Table S2** of mean(SD) for coherence, PACz, DFA and fE/I.

| ***Name of state*** | ***Coherence*** | ***PACz***  *P*hase freq. range | ***DFA***  ***Peak freq., Hurst exponent*** | ***fE/I***  ***Max.***  ***Min.*** |
| --- | --- | --- | --- | --- |
| ***A1 (awake, active)*** | ***9.4 Hz (1.3)****,*  0.67 (0.18) | ***6.9 Hz (4.2)****,*  *21 (14)* | ***P1 4.4 Hz (1.5)****, H 0.76 (0.09)*  ***P2 11 Hz (2.3)****, H 0.79 (0.074)* | ***7.9 Hz (4.5)****, 0.95 (0.20)*  ***34 Hz (26)****, 0.68 (0.27)* |
| ***A2 (awake, not necessarily active)*** | ***7.4 Hz (1.4)****,*  *0.62 (0.18)* | ***7.8 Hz (3.4)****,*  *9.8 (8.4)* | ***P1 4.3 Hz (1.9)****, H 0.78 (0.078)*  ***P2 11 Hz (3.2)****, H 0.76 (0.06)* | ***6.1 Hz (7.5)****, 1.0 (0.1)*  ***35 Hz (21)****, 0.61 (0.15)* |
| ***A3 (resting state, inactive)*** | ***9.4 Hz (1.0)****,*  *0.68 (0.18)* | ***7.7 Hz (3.7)****,*  *8.9 (9.4)* | ***P 12 Hz (2.6)****, H 0.75 (0.14)* | ***8.8 Hz (7.9)****, 1.1 (0.3)*  ***33 Hz (21)****, 0.35 (0.24)* |
| ***REM (sleep)*** | ***8.3 Hz (0.82)****,*  *0.71 (0.16)* | ***7.9 Hz (2.3)****,*  *10 (8.3)* | ***P1 5 Hz (1.8)****, H 0.76 (0.071)*  ***P2 11 Hz (3.2)****, H 0.77 (0.076)* | ***6.7 Hz (6.8)****, 1.1 (0.19)*  ***25 Hz (19)****, 0.51 (0.18)* |
| ***nREM1 (slow-wave sleep)*** | ***1.8 Hz (0.92)****,*  *0.69 (0.17)* | ***3.0 Hz (2.4)****,*  *22 (11)* | ***P 2.8 Hz (2.6)****, H 0.69 (0.073)* | ***4.7 Hz (4.3)****, 1.3 (0.18)*  ***28 Hz (23)****, 0.69 (0.3)* |
| ***nREM2 (slightly faster than slow-wave)*** | ***1.8 Hz (1.0)****,*  *0.65 (0.16)* | ***4.9 Hz (3.7)****,*  *18 (8.3)* | ***P 2.2 Hz (1.7)****, H 0.80 (0.091)* | ***3.5 Hz (1.9)****, 1.2 (0.11)*  ***27 Hz (22)****, 0.58 (0.12)* |
| ***nREM3 (includes sleep spindles)*** | ***1.4 Hz (1.1)****,*  *0.59 (0.15)* | ***8.1 Hz (3.6)****,*  *10 (7.2)* | ***P1 2.8 Hz (1.4)****, H 0.99 (0.12)*  ***P2 12 Hz (3)****, H 0.96 (0.12)* | ***5.3 Hz (8)****, 1.0 (0.12)*  ***26 Hz (17)****, 0.50 (0.13)* |

# Pcdh9 35 animals (all male), four genotypes, two time points (8 weeks, 22 weeks)


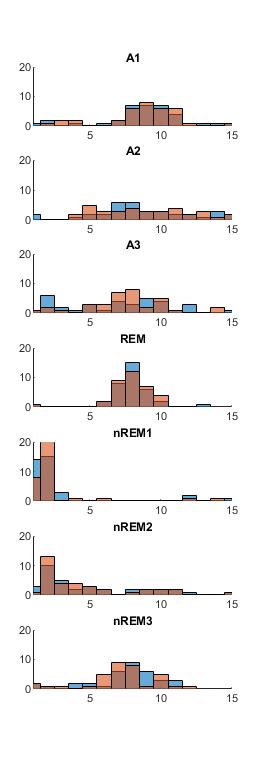

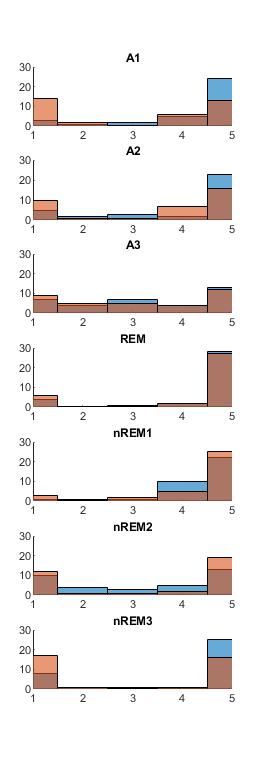

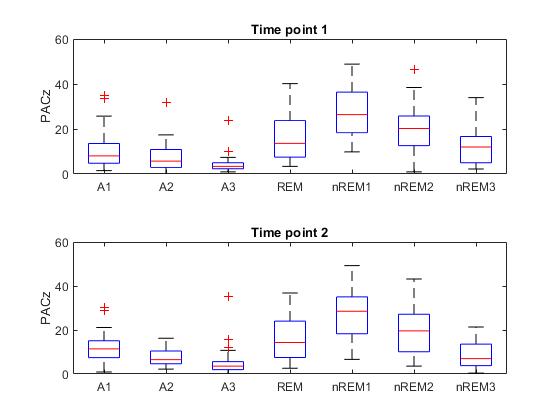


**A**

**B**

**C**

**Figure S8** PACz distribution. Blue: 8 weeks (time point 1), red: 22 weeks (time point 2).

**Table S3** displaying the mean (SD) of coherence, PACz, DFA and fE/I for all Pcdh9 data is pooled across genotype and time.

| ***Name of state*** | ***Coherence*** | ***PACz***  *P*hase freq. range | ***DFA***  ***Peak freq., Hurst exponent*** | ***fE/I***  ***Max.***  ***Min.*** |
| --- | --- | --- | --- | --- |
| ***A1 (awake, active)*** | ***8.3 Hz (1.3)****,*  0.77 (0.17) | ***8.7 Hz (2.9)****,*  *11 (7.6)* | ***P1 4.5 Hz (0.85)****, H 0.89 (0.07)*  ***P2 10 Hz (2.5)****, H 0.82 (0.079)* | ***7.8 Hz (8.1)****, 1.0 (0.16)*  ***20 Hz (14)****, 0.72 (0.19)* |
| ***A2 (awake, not necessarily active)*** | ***7.0 Hz (1.3)****,*  *0.73 (0.18)* | ***8.8 Hz (3.4)****,*  *7.3 (5.4)* | ***P1 4.1 Hz (1.9)****, H 0.76 (0.077)*  ***P2 11 Hz (3.1)****, H 0.75 (0.073)* | ***6.7 Hz (9.5)****, 1.0 (0.16)*  ***20 Hz (15)****, 0.65 (0.11)* |
| ***A3 (resting state, inactive)*** | ***8.3 Hz (1.2)****,*  *0.77 (0.16)* | ***7.2 Hz (3.3)****,*  *4.6 (5.2)* | ***P 12 Hz (3)****, H 0.83 (0.22)* | ***6.8 Hz (11)****, 1.2 (0.34)*  ***24 Hz (14)****, 0.27 (0.21)* |
| ***REM (sleep)*** | ***7.9 Hz (0.54)****,*  *0.82 (0.15)* | ***7.9 Hz (1.7)****,*  *16 (9.7)* | ***P1 5 Hz (1.7)****, H 0.73 (0.062)*  ***P2 12 Hz (3.2)****, H 0.74 (0.065)* | ***10 Hz (10)****, 1.2 (0.12)*  ***29 Hz (17)****, 0.58 (0.15)* |
| ***nREM1 (slow-wave sleep)*** | ***1.8 Hz (0.76)****,*  *0.79 (0.16)* | ***2.6 Hz (3.0)****,*  *28 (11)* | ***P 1.9 Hz (2.3)****, H 0.71 (0.073)* | ***5.2 Hz (8.3)****, 1.2 (0.14)*  ***19 Hz (13)****, 0.73 (0.21)* |
| ***nREM2 (slightly faster than slow-wave)*** | ***1.5 Hz (0.85)****,*  *0.76 (0.17)* | ***4.9 Hz (3.6)****,*  *20 (10)* | ***P 1.5 Hz (1.4)****, H 0.78 (0.079)* | ***4.7 Hz (7.8)****, 1.1 (0.082)*  ***19 Hz (13)****, 0.56 (0.076)* |
| ***nREM3 (includes sleep spindles)*** | ***1.5 Hz (1.1)****,*  *0.7 (0.18)* | ***7.3 Hz (2.5)****,*  *10 (7.0)* | ***P1 3 Hz (1.4)****, H 0.94 (0.094)*  ***P2 13 Hz (1.8)****, H 0.93 (0.082)* | ***4 Hz (8)****, 0.95 (0.085)*  ***18 Hz (9.8)****, 0.44 (0.085)* |

# Nrxn1 23 animals (all male), three genotypes, two timepoints (1=8 weeks, 2= 22 weeks)


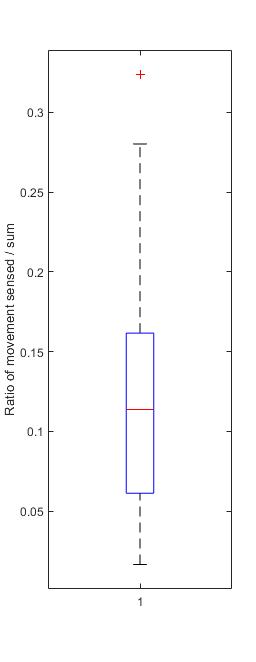


**Figure S9** Accuracy of movement sensors.


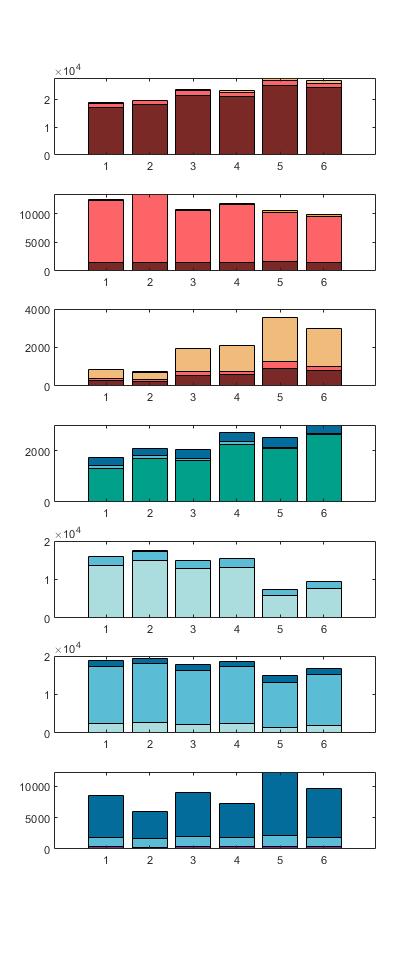

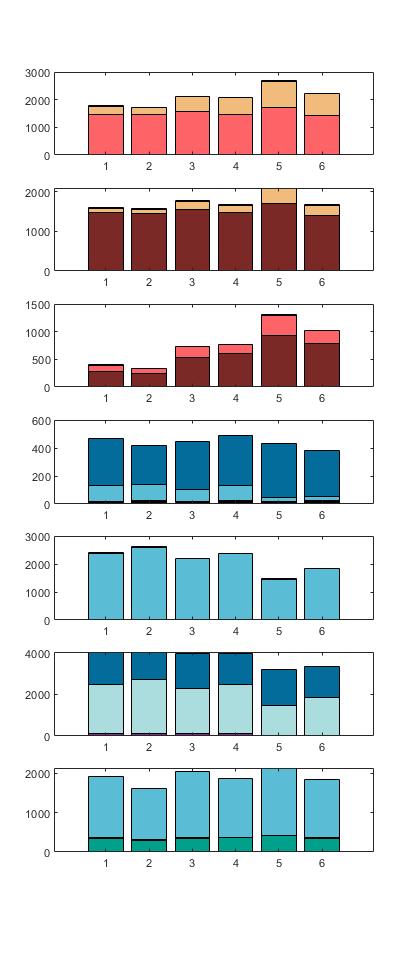


**A**

**B**

**Figure S10** A) Pairs of epoch labels. 1; WT1, 2; WT2, 3; Het1, 4; Het2, 5; Hom1, 6; Hom2. Bordeaux; a1, pink; a2, peach; a3, turquois; REM, light blue; nREM1, blue; nREM2, dark blue; nREM3. B) bar plots of the transitions from the vigilance state. Major transitions from both a2 and a3 are to a1. REM transitions mostly to nREM3 and nREM2. nREM1 transitions almost exclusively to nREM2. nREM2 transitions to both nREM1 and nREM3. nREM3 transitions mostly to nREM2 and some to REM.


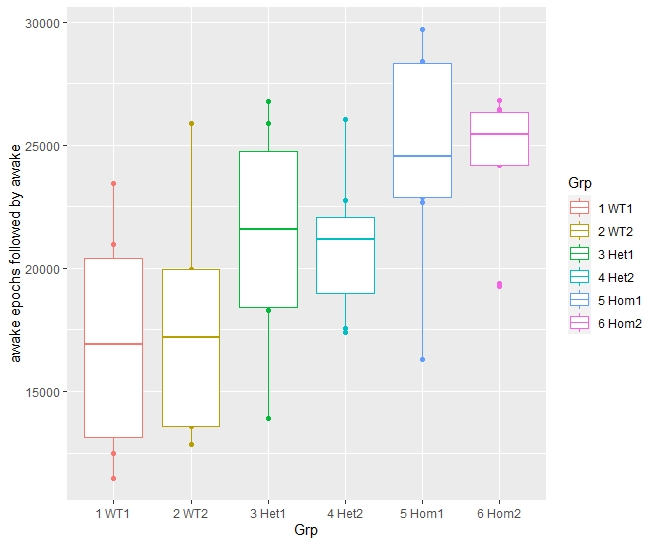

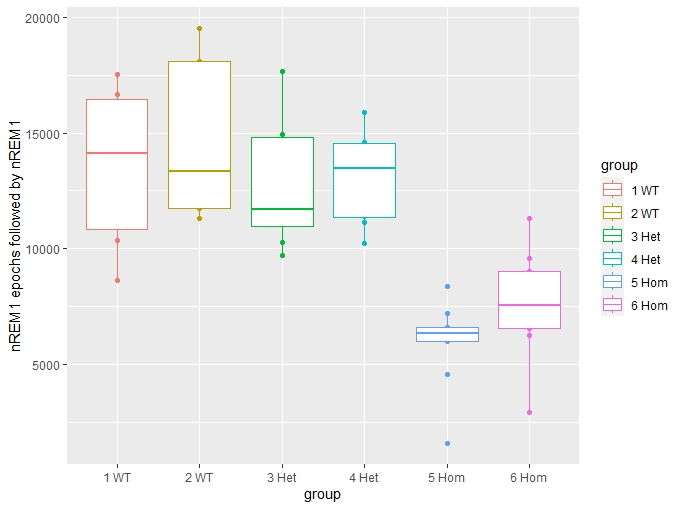


**A**

**B**

**Figure S11** A) Epochs labelled as δ awake followed by another δ awake epochs over the course of 24 h recorded at 1; 8 weeks and 2; 22 weeks. Grp = group B) nREM1 epochs followed by nREM1 epochs.
